# Chytridiomycosis-induced mortality in a threatened anuran

**DOI:** 10.1371/journal.pone.0241119

**Published:** 2020-11-06

**Authors:** Andrea J. Adams, Allan Pessier, Peggy Cranston, Robert L. Grasso

**Affiliations:** 1 Yosemite National Park, El Portal, California, United States of America; 2 Earth Research Institute, University of California Santa Barbara, Santa Barbara, California, United States of America; 3 Department of Veterinary Microbiology and Pathology, College of Veterinary Medicine, Washington State University, Pullman, Washington, United States of America; 4 Mother Lode Field Office, U.S. Bureau of Land Management, Fair Oaks, California, United States of America; University of South Dakota, UNITED STATES

## Abstract

Effectively planning conservation introductions involves assessing the suitability of both donor and recipient populations, including the landscape of disease risk. Chytridiomycosis, caused by the fungal pathogen *Batrachochytrium dendrobatidis* (Bd), has caused extensive amphibian declines globally and may hamper reintroduction attempts. To determine Bd dynamics in potential source populations for conservation translocations of the threatened California red-legged frog (*Rana draytonii*) to Yosemite National Park, we conducted Bd sampling in two populations in the foothills of the Sierra Nevada Mountains, California, U.S.A. At one of two sites, we observed lethally high Bd loads in early post-metamorphic life stages and confirmed one chytridiomycosis-induced mortality, the first such report for this species. These results informed source population site selection for subsequent *R*. *draytonii* conservation translocations. Conservation efforts aimed at establishing new populations of *R*. *draytonii* in a landscape where Bd is ubiquitous can benefit from an improved understanding of risk through disease monitoring and *ex situ* infection studies.

## Introduction

Successful conservation translocations hinge on adequate preparation and planning; along with habitat suitability and source population stability, disease susceptibility is a critical consideration [1–3]. The fungal pathogen *Batrachochytrium dendrobatidis* (hereafter Bd), the causative agent of the disease chytridiomycosis, is a primary cause of widespread amphibian declines globally [4–6], and can hamper amphibian reintroduction attempts [7].

The largest anuran native to the western United States (Wright and Wright 1949), the California red-legged frog (*R*. *draytonii*) has been extirpated from >70% of its former range, prompting calls for reintroduction feasibility studies [8]. Listed as threatened under the U.S. Endangered Species Act since 1996, *R*. *draytonii* was originally threatened by overharvest in the nineteenth and early twentieth centuries [9, 10]. Since the rapid urbanization of California, the species has declined due to habitat loss, pesticides, and introduced predators [11–14]. The extent to which disease may have contributed to *R*. *draytonii* decline is unknown; however, higher Bd prevalence in the species has been associated with decreased survival [15].

No chytridiomycosis-induced mortality has been recorded for *R*. *draytonii*, though the closely-related congeners *Rana muscosa*, *Rana sierrae*, and *Rana boylii* have all experienced chytridiomycosis-induced die-offs in California [14, 16]. California red-legged frogs are generally presumed tolerant of Bd because they persist in areas where Bd is present [17, 18]. Bd prevalence in wild *R*. *draytonii* populations in California and Mexico ranges from 37% to 68% [14, 18–20], but data regarding *R*. *draytonii* Bd susceptibility are sparse. In the only published laboratory study of Bd infection in *R*. *draytonii* to date, Bd-positive metamorphs of unknown Bd load with wild-caught Bd infection did not present with morbidity or mortality during 18 months of laboratory observation [21]. In *ex situ R*. *draytonii* infection trials, individuals did not experience chytridiomycosis-induced morbidity or mortality [22], indicating that adults may have some measure of innate and adaptive immunity to Bd.

Among species, host responses to Bd infection are highly variable, ranging from lethal susceptibility to tolerance, and, in some cases, complete resistance to infection [23–25]. Within-species disease outcome is also variable, ranging from disease-induced localized extirpations to infection tolerance, population persistence, and recovery [26, 27]. Variation in host Bd resistance and tolerance can be influenced by host environment [28]; behavior [19]; genetically mediated immune factors [29, 30] or the lineage of Bd infecting the host [31, 32]. Because reconstruction of the immune system occurs during metamorphosis, immunosuppression can make recently metamorphosed individuals particularly vulnerable to disease [33–35].

Donor population selection and the life stage of introduced individuals may be essential factors in determining reintroduction success when conducting conservation translocations in a landscape where Bd is essentially ubiquitous [2]. Here, we report on Bd sampling at two candidate *R*. *draytonii* source populations to evaluate their suitability for conservation translocations.

## Materials and methods

### Ethics statement

Rana draytonii capture and handling were conducted under permits issued by the U.S. Fish and Wildlife Service (TE-86906B-0), California Department of Fish and Wildlife (SC-5130), and Yosemite National Park (YOSE-2015-SCI-129 and YOSE-2016-SCI-101).

### Study sites

**Fig 1:**
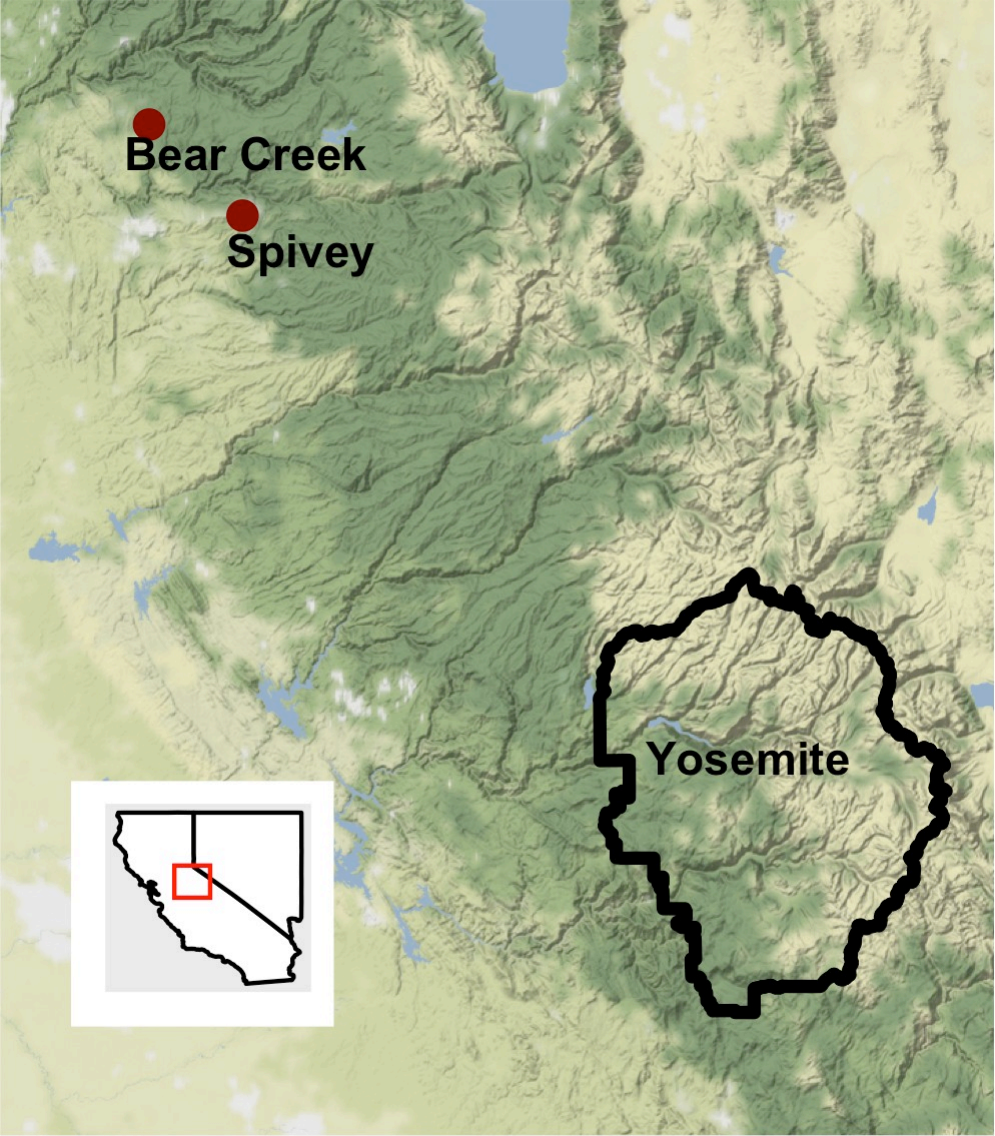
Map of the study area. The map was generated in R [67] using the “ggmap” [78] and “ggplot2” [79] packages with map tiles by Stamen Design (www.stamen.com) and data by OpenStreetMap, under ODbL, under CC BY 3.0 (creativecommons.org/licenses/by/3.0/).

The Sierra Nevada Mountains have become an epicenter of amphibian decline studies [13, 27, 36–44]. Extant Sierra Nevada *R*. *draytonii* populations are scarce [45] and rarely accessible for proactive conservation efforts, primarily due to their presence on privately owned lands. We identified two candidate *R*. *draytonii* source populations for translocations: Bear Creek Pond (790 m elevation) and Spivey Pond (975 m elevation), in El Dorado County, California, USA, which are artificial creek impoundments fed by headwater springs (Fig 1). When the Spivey Pond *R*. *draytonii* population was discovered in 1997, it was the first report for the species in the Sierra Nevada in nearly 25 years, and is currently one of only six known populations of the species in the mountain range [46]. In 1998, the site was conserved as part of a 20-hectare parcel and became public land when it was sold to the U.S. Bureau of Land Management [46]. Prior to this study, Spivey Pond had only been sampled for Bd once: two adults were sampled in 2009, and although quantitative data are not available, a “strong Bd-positive signal” was detected on one of two adult frogs [47].

Effective population sizes at Bear Creek are the largest among five Sierra Nevada foothill populations sampled for mtDNA analysis (N_e_ = 19.67–41.23) [48]. Bear Creek and Spivey Pond *R*. *draytonii* share a common mtDNA haplotype, indicating connectivity between the two sites at some time in the past, though there does not appear to be any contemporary gene flow [48]. Both ponds have similar amphibian species assemblages, including *R*. *draytonii*, *Anaxyrus boreas halophilus* (California toad), and *Pseudacris regilla* (Pacific chorus frog); however, Spivey also has American bullfrogs (*Rana catesbeiana*; hereafter “bullfrogs”) and Sierra newts (*Taricha sierrae*), whereas Bear Creek does not.

### Study species

In the Sierra Nevada, *R*. *draytonii* is limited to small, isolated populations in the northern portion of its range with restricted gene exchange [48]. The species is present in fewer than 30% of historical localities, most of which have very small population sizes [48]. In Yosemite National Park (Yosemite), *R*. *draytonii* had been extirpated prior to the translocation project and were not currently known to occur within 160 km as evidenced by visual encounter surveys and environmental DNA. However, the recent eradication of bullfrogs from Yosemite Valley has made the establishment of California red-legged frogs in Yosemite possible for the first time in over 60 years [49, 50].

American bullfrogs (*Rana catesbeiana* after Yuan, Zhou [51]; hereafter “bullfrogs”) were introduced to California from the eastern USA in the 19^th^ and early 20^th^ centuries. They are an invasive predator and competitor of many native aquatic species in the western USA and globally, and have been implicated in *R*. *draytonii* declines [13, 52–61]. Bullfrogs are susceptible to chytridiomycosis infection but appear tolerant of most Bd strains, making them suitable vectors and reservoir hosts for the pathogen in the wild [19, 62, 63]. Bullfrogs were first observed at Spivey Pond in 2000, and in 2003, the pond was drained in order to reduce the population of the species, which has an obligate two-year tadpole stage [64]. Measures have also been taken to reduce adult bullfrogs at the site, including egg mass removal and direct lethal taking.

### Field surveys and laboratory analyses

We nocturnally surveyed for all lifestages of *R*. *draytonii* at both populations, detecting adults via eye shine using 200 lumen LED flashlights, and detecting subadults (<50 mm snout-vent length (SVL)) opportunistically during both daytime and nighttime site visits. We surveyed Bear Creek and Spivey in June and October 2016, and conducted additional surveys at Bear Creek in October 2015 and June 2017. We captured individual adults and subadults with fresh pairs of nitrile gloves and sampled them for Bd following standardized protocols using a rayon-tipped swab (Hyatt et al. 2007). We used quantitative polymerase chain reaction (qPCR) to detect Bd DNA following Boyle et al. (2004). We measured Bd infection intensity (Bd load) in terms of zoospore equivalents (ZE), calculated by multiplying the genomic equivalents by 80 to account for the dilution factor in qPCR sample preparation necessitated by the use of standard DNA extraction methods for swabs collected from live animals [65]. We used a Welch t-test [66] on log-transformed Bd values (ZE) to compare mean Bd loads of adults and subadults on one sample date. Statistical analyses were conducted and all figures were created using R [67].

We collected one subadult (24 mm SVL) from Spivey Pond with lethargy and loss of righting reflex to determine the cause of morbidity and eventual mortality. We fixed the frog whole in ethanol, post-fixed in 10% neutral buffered formalin, and decalcified in hydrochloric acid. After decalcification, the body was serially sectioned and processed routinely for histologic examination in two paraffin blocks [68].

## Results and discussion

**Fig 2:**
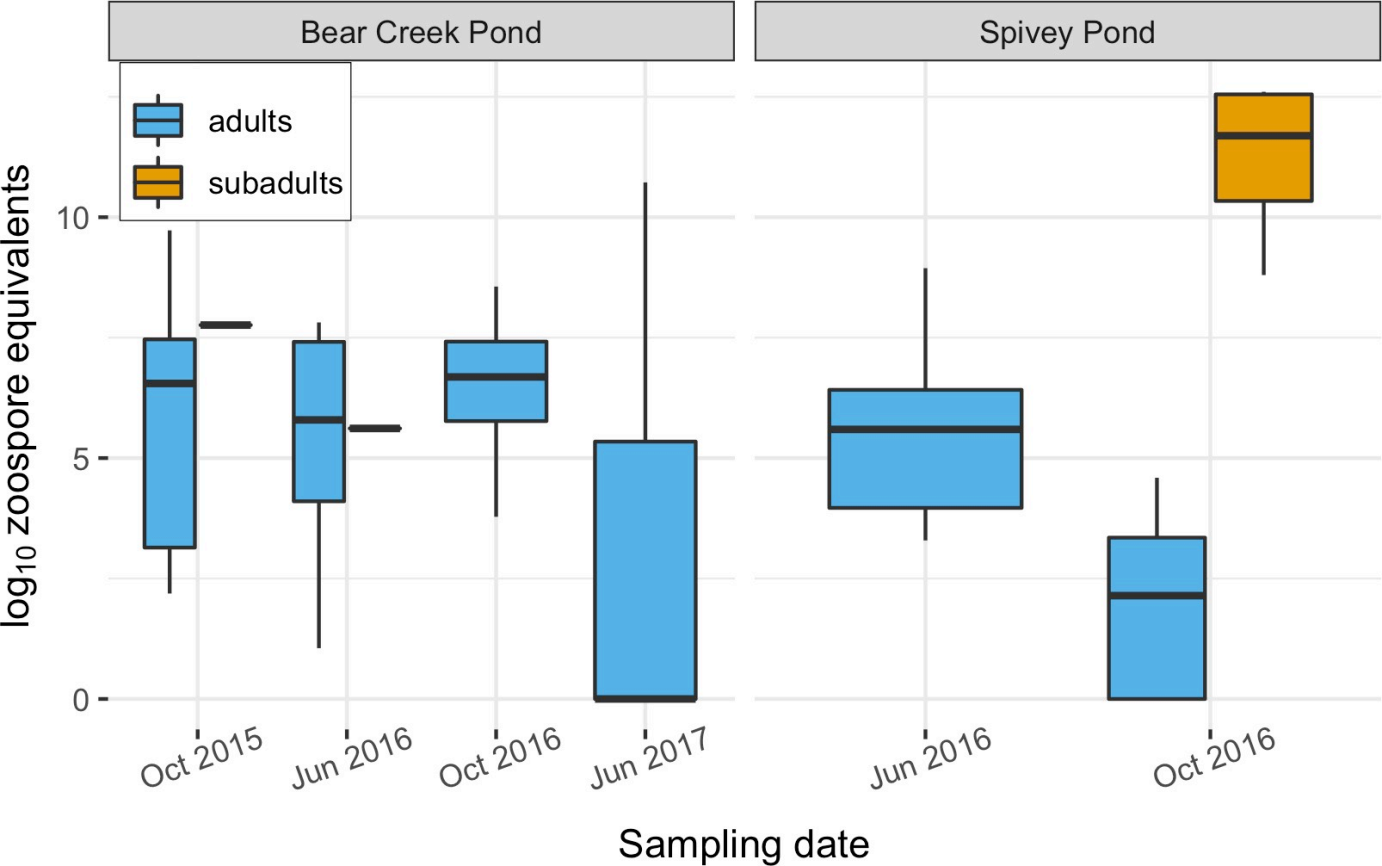
*Batrachochytrium dendrobatidis* (Bd) loads in Bd-positive California red-legged frogs (*Rana draytonii*) at two translocation donor populations in El Dorado County, California, U.S.A. Box widths are proportional to sample size, bold horizontal lines within each boxplot indicate the median, boxes show the interquartile (IQ) range, and whiskers show the range within 1.5 times the IQ range.

We sampled 63 *R*. *draytonii* individuals (57 adults and 6 subadults) for Bd. Bd prevalence at Bear Creek was 85% (n = 41, 95% CI 71–94) and Spivey Pond was 86% (n = 22, 95% CI 65–97)—among the highest prevalence reported for this species [14, 18, 69]. At Spivey Pond in October 2016—the only date that more than one subadult was captured—subadult Bd loads were significantly higher than those of adults (Welch’s t(5), t = -7.6, p = 0.0006; Fig 2). The moribund subadult collected at Spivey had the highest Bd load (297,700 ZE) of all animals sampled.

**Fig 3:**
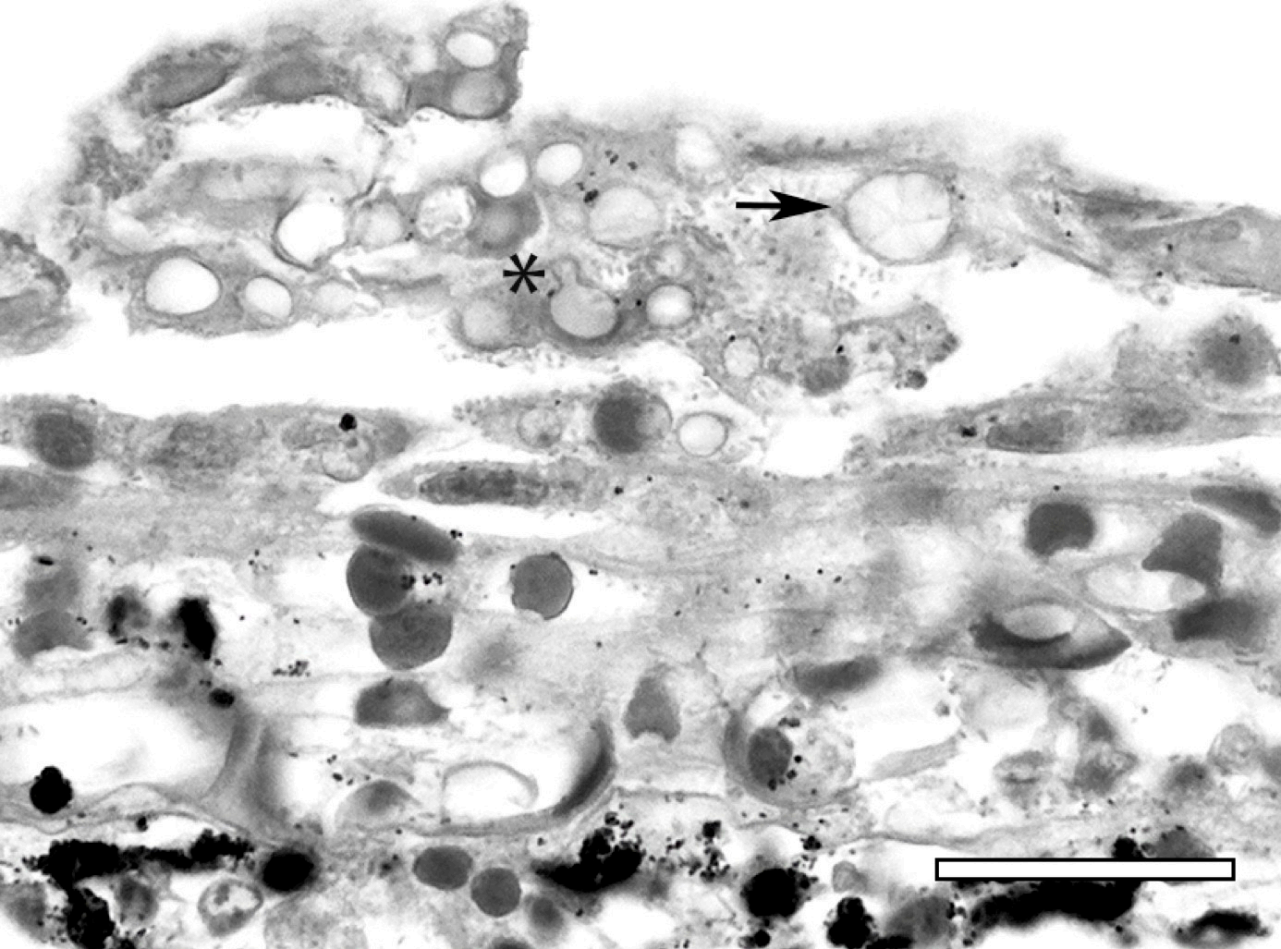
Histologic findings from a California red-legged frog (*Rana draytonii*) with chytridiomycosis. Marked hyperkeratosis with numerous empty chytrid fungal thalli. Characteristic thallus forms include zoosporangia with prominent discharge tubes (asterisk) and internally septate colonial thalli (arrow). Bar = 30 microns.

Histologic findings of the moribund subadult from Spivey were consistent with clinically significant (lethal) chytridiomycosis caused by Bd infection. Examination demonstrated diffuse epidermal hyperplasia and orthokeratotic hyperkeratosis with myriad intracorneal chytrid-type fungal thalli (Fig 3). Most chytrid thalli were empty from previous discharge of zoospores but forms included flask-shaped zoosporangia with prominent discharge tubes and internally septate colonial thalli consistent with the genus *Batrachochytrium*. The distribution of skin lesions and number of fungal thalli present was consistent with lethal chytridiomycosis in other anuran species [39, 70–72]. There was no histologic evidence of another contributory disease process (e.g. ranavirus infection).

In addition to the moribund frog collected for histological examination, two subadults with loads considered lethally high in other ranid species [>10,000 ZE; 73] did not exhibit symptoms of chytridiomycosis in the field and were therefore not collected. Outward symptoms of the disease are not typically observed until very late stage morbidity; therefore, observing lethally infected frogs when they are symptomatic is rare [74].

**Fig 4:**
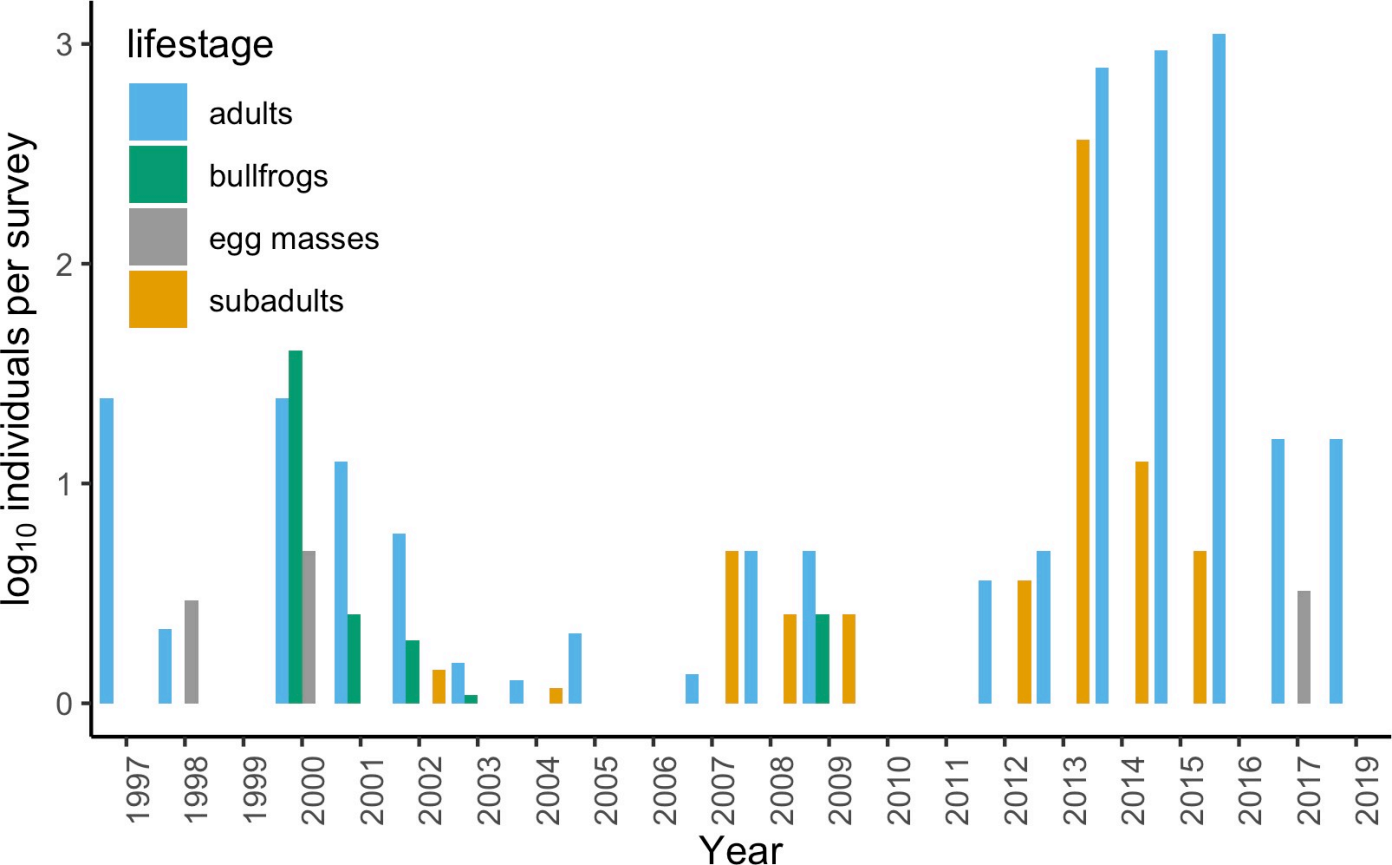
Twenty-two-year monitoring data for Spivey Pond collected and curated by the U.S. Bureau of Land Management. Lifestages are for California red-legged frogs (*Rana draytonii*); bullfrogs are non-native American bullfrogs (*Rana catesbeiana*).

Twenty-two years of Spivey Pond *R*. *draytonii* monitoring indicate that life stages and egg mass counts were variable across years (Fig 4). Increased detection of frogs in 2014, 2015, and 2016 could have resulted from drought, which concentrates frogs into smaller aquatic habitats [19]. Chytridiomycosis mortalities in a California congener (*R*. *boylii*) have been attributed to lower flow rates during drought conditions, which may also increase pathogen transmission opportunities from crowding of aquatic habitats [19]. The presence of a bullfrog Bd vector and reservoir host can increase pathogen burden in native California ranids [19]. Bullfrogs have historically been present at Spivey, but not Bear Creek; however, bullfrogs have not been observed at Spivey since 2009 (Fig 4).

Subadults were encountered less frequently than adults, constituting only 17% of Spivey samples and 5% of Bear Creek samples collected (Fig 2). In addition to subadult Bd mortality, Sierra newt (*Taricha sierrae*) predation may keep subadult *R*. *draytonii* densities low at Spivey as compared to Bear Creek. A predator of *R*. *draytonii*, *T*. *sierrae* and can account for up to 90% of embryo mortality (Calef 1973; Licht 1974). At Spivey, *T*. *sierrae* have been observed on egg masses in high abundance (>100), presumably waiting for tadpoles to hatch; *T*. *sierrae* is not present at Bear Creek. At Bear Creek, the adult *R*. *draytonii* population is larger (>100 frogs), and in a typical year 25–35 egg masses are observed. Higher densities at Bear Creek may increase *R*. *draytonii* cannibalism and reduce the subadult population [75].

## Conclusions

This is the first report of chytridiomycosis-induced mortality in *R*. *draytonii*. We observed adult Bd loads well below those considered lethal in other ranids [19, 73], but observed extremely high loads in subadults (Fig 2), including one moribund individual. Though our sample sizes are too small to definitively conclude that subadults are more likely to be infected compared to adults, our observation is consistent with high Bd loads and mortality in the subadult stage of other Bd-susceptible ranid species, including *R*. *muscosa* and *R*. *boylii* in California [16, 19], and *R*. *onca* in Nevada [35]. The subadult in close proximity to the moribund frog that exhibited Bd loads on the same order of magnitude (>270,000 ZE) but had no outward symptoms highlights the importance of Bd sampling and qPCR detection to determine degree of infection rather than behavioral observations alone.

Though we do not have ample evidence to conclude that chytridiomycosis is a major source of mortality in *R*. *draytonii*, our finding of a link between Bd infection and mortality has been a consideration in the ongoing conservation translocation project in Yosemite. More broadly, this report should be considered when reintroductions or other elements called for in the recovery plan for this threatened species—such as mitigation banking—are undertaken. *Rana draytonii* populations with higher Bd prevalence exhibit lower survivorship [15], and mathematical models largely suggest that the post-metamorphic juvenile life stage can be a disproportionately essential driver of amphibian population dynamics [76]. Future work should use *ex situ* Bd inoculations of early post-metamorphic *R*. *draytonii* to determine how commonly juvenile Bd mortality can occur.

National parks are often considered refugia for species that are unable to persist in the face of threats outside of protected areas [77], and anthropogenic stressors outside of Yosemite—such as invasive predators and competitors—currently limit California red-legged frog reintroduction efforts. Disease is a threat indifferent to geopolitical boundaries, and thus the need for reintroduction feasibility research both inside and outside of protected areas is imperative. In a landscape where pathogens—such as Bd—are ubiquitous, diligent monitoring can improve managers’ understanding of disease risk. We recommend that *ex situ* Bd exposure studies be conducted with *R*. *draytonii* to further examine the susceptibility of this species in the vulnerable early post-metamorphic life stage.

## Supporting information

S1 Data (PDF)

S2 Data (PDF)
