# Supplementary material for: Chytridiomycosis-induced mortality in a threatened anuran
Source: PLoS One. 2020 Nov 6;15(11):e0241119. doi: 10.1371/journal.pone.0241119 (PMC7647137; doi:10.1371/journal.pone.0241119)
Supplement: S1 Data — (PDF) [file pone.0241119.s001.pdf]

site	date	stage	ze
Spivey Pond	10/4/16	Adult	0
Spivey Pond	10/4/16	Adult	0
Spivey Pond	10/4/16	Adult	0
Bear Creek Pond	6/23/17	Adult	0
Bear Creek Pond	6/23/17	Adult	0
Bear Creek Pond	6/23/17	Adult	0
Bear Creek Pond	6/23/17	Adult	0
Bear Creek Pond	6/23/17	Adult	0
Bear Creek Pond	6/23/17	Adult	0
Bear Creek Pond	10/7/15	Adult	7.92
Bear Creek Pond	10/7/15	Adult	10.91
Bear Creek Pond	10/7/15	Adult	22.16
Bear Creek Pond	10/7/15	Adult	31.21
Bear Creek Pond	10/7/15	Adult	697.65
Bear Creek Pond	10/7/15	Adult	1155
Bear Creek Pond	10/7/15	Adult	1744.09
Bear Creek Pond	10/7/15	Subadult	2347.58
Bear Creek Pond	10/7/15	Adult	4503.65
Bear Creek Pond	10/7/15	Adult	16766.16
Bear Creek Pond	6/13/16	Adult	1.87
Bear Creek Pond	6/13/16	Adult	14.16
Bear Creek Pond	6/13/16	Adult	59.51
Bear Creek Pond	6/13/16	Subadult	273.78
Bear Creek Pond	6/13/16	Adult	288.28
Bear Creek Pond	6/13/16	Adult	326.85
Bear Creek Pond	6/13/16	Adult	1097.27
Bear Creek Pond	6/13/16	Adult	1655.71
Bear Creek Pond	6/13/16	Adult	1790.49
Bear Creek Pond	6/13/16	Adult	2480.59
Spivey Pond	6/14/16	Adult	25.85
Spivey Pond	6/14/16	Adult	35.23
Spivey Pond	6/14/16	Adult	35.62
Spivey Pond	6/14/16	Adult	75.11
Spivey Pond	6/14/16	Adult	244.89
Spivey Pond	6/14/16	Adult	268.23
Spivey Pond	6/14/16	Adult	300.95
Spivey Pond	6/14/16	Adult	532.32
Spivey Pond	6/14/16	Adult	700.84
Spivey Pond	6/14/16	Adult	1590.24
Spivey Pond	6/14/16	Adult	7652.49
Spivey Pond	10/4/16	Adult	7.54
Spivey Pond	10/4/16	Adult	22.93
Spivey Pond	10/4/16	Adult	32.86

Bear Creek Pond	10/4/16	Adult	42.87
Spivey Pond	10/4/16	Adult	97.61
Bear Creek Pond	10/4/16	Adult	230.79
Bear Creek Pond	10/4/16	Adult	291.54
Bear Creek Pond	10/4/16	Adult	348.64
Bear Creek Pond	10/4/16	Adult	621.25
Bear Creek Pond	10/4/16	Adult	798.91
Bear Creek Pond	10/4/16	Adult	863.11
Bear Creek Pond	10/4/16	Adult	1149.29
Bear Creek Pond	10/4/16	Adult	2413.85
Bear Creek Pond	10/4/16	Adult	2976.91
Bear Creek Pond	10/4/16	Adult	5219.68
Spivey Pond	10/4/16	Subadult	6624.4
Spivey Pond	10/4/16	Subadult	51535.18
Spivey Pond	10/4/16	Subadult	277119.69
Spivey Pond	10/4/16	Subadult	297783.81
Bear Creek Pond	6/23/17	Adult	110.16
Bear Creek Pond	6/23/17	Adult	256.89
Bear Creek Pond	6/23/17	Adult	482.92
Bear Creek Pond	6/23/17	Adult	45339
