# Supplementary material for: Chytridiomycosis-induced mortality in a threatened anuran
Source: PLoS One. 2020 Nov 6;15(11):e0241119. doi: 10.1371/journal.pone.0241119 (PMC7647137; doi:10.1371/journal.pone.0241119)
Supplement: S2 Data — (PDF) [file pone.0241119.s002.pdf]

year	lifestage	indivs_survey
1997	bullf_surv	0.00
1998	bullf_surv	0.00
1999	bullf_surv	0.00
2000	bullf_surv	5.00
2001	bullf_surv	0.50
2002	bullf_surv	0.33
2003	bullf_surv	0.04
2004	bullf_surv	0.00
2005	bullf_surv	0.00
2006	bullf_surv	0.00
2007	bullf_surv	0.00
2008	bullf_surv	0.00
2009	bullf_surv	0.50
2010	bullf_surv	0.00
2011	bullf_surv	0.00
2012	bullf_surv	0.00
2013	bullf_surv	0.00
2014	bullf_surv	0.00
2015	bullf_surv	0.00
2016	bullf_surv	0.00
2017	bullf_surv	0.00
2019	bullf_surv	0.00
1997	eggm_surv	0.00
1998	eggm_surv	0.60
1999	eggm_surv	0.00
2000	eggm_surv	1.00
2001	eggm_surv	0.00
2002	eggm_surv	0.00
2003	eggm_surv	0.00
2004	eggm_surv	0.00
2005	eggm_surv	0.00
2006	eggm_surv	0.00
2007	eggm_surv	0.00
2008	eggm_surv	0.00
2009	eggm_surv	0.00
2010	eggm_surv	0.00
2011	eggm_surv	0.00
2012	eggm_surv	0.00
2013	eggm_surv	0.00
2014	eggm_surv	0.00
2015	eggm_surv	0.00
2016	eggm_surv	0.00
2017	eggm_surv	0.67
2019	eggm_surv	0.00
1997	ju_surv	0.00
1998	ju_surv	0.00
1999	ju_surv	0.00
2000	ju_surv	0.00
2001	ju_surv	0.00
2002	ju_surv	0.17

2003	ju_surv	0.00
2004	ju_surv	0.07
2005	ju_surv	0.00
2006	ju_surv	0.00
2007	ju_surv	1.00
2008	ju_surv	0.50
2009	ju_surv	0.50
2010	ju_surv	0.00
2011	ju_surv	0.00
2012	ju_surv	0.75
2013	ju_surv	12.00
2014	ju_surv	2.00
2015	ju_surv	1.00
2016	ju_surv	0.00
2017	ju_surv	0.00
2019	ju_surv	0.00
1997	ad_surv	3.00
1998	ad_surv	0.40
1999	ad_surv	0.00
2000	ad_surv	3.00
2001	ad_surv	2.00
2002	ad_surv	1.17
2003	ad_surv	0.20
2004	ad_surv	0.11
2005	ad_surv	0.38
2006	ad_surv	0.00
2007	ad_surv	0.14
2008	ad_surv	1.00
2009	ad_surv	1.00
2010	ad_surv	0.00
2011	ad_surv	0.00
2012	ad_surv	0.75
2013	ad_surv	1.00
2014	ad_surv	17.00
2015	ad_surv	18.50
2016	ad_surv	20.00
2017	ad_surv	2.33
2019	ad_surv	2.33
